# Environmental Microbial Community Proteomics: Status, Challenges and Perspectives

**DOI:** 10.3390/ijms17081275

**Published:** 2016-08-05

**Authors:** Da-Zhi Wang, Ling-Fen Kong, Yuan-Yuan Li, Zhang-Xian Xie

**Affiliations:** State Key Laboratory of Marine Environmental Science, College of the Environment and Ecology, Xiamen University, Xiamen 361102, China; konglingfen513@gmail.com (L.-F.K.); liyuanyuan9215@126.com (Y.-Y.L.); xiezhangxian@163.com (Z.-X.X.)

**Keywords:** microbes, proteomics, community proteomics, metaproteomics

## Abstract

Microbial community proteomics, also termed metaproteomics, is an emerging field within the area of microbiology, which studies the entire protein complement recovered directly from a complex environmental microbial community at a given point in time. Although it is still in its infancy, microbial community proteomics has shown its powerful potential in exploring microbial diversity, metabolic potential, ecological function and microbe-environment interactions. In this paper, we review recent advances achieved in microbial community proteomics conducted in diverse environments, such as marine and freshwater, sediment and soil, activated sludge, acid mine drainage biofilms and symbiotic communities. The challenges facing microbial community proteomics are also discussed, and we believe that microbial community proteomics will greatly enhance our understanding of the microbial world and its interactions with the environment.

## 1. Introduction

Microorganisms and their activities are of critical importance to virtually all biological systems on our planet. The composition and structure of microbial communities are diverse over a wide range of environments [1]. Moreover, microbial metabolic activities and cellular physiology frequently fluctuate along with environmental changes [2]. The ubiquity and complexity of microbial communities have been extensively studied for decades [3]; however, their ecological functions in the environment and their response to various environmental drivers has gained increasing attention in recent years.

Microbial community proteomics (also termed metaproteomics), which characterizes all the proteins expressed at a given time within an ecosystem, plays a key role in exploring microbial functionality [4]. Investigation of the protein expression of a microbial community enables an unprecedented view of the adaptive responses of microbes to environmental stimuli or their interactions with other organisms or host cells [5]. Studies of the microbial community in natural environments have expanded our knowledge of microbial functions, such as nutrient cycling, mutualistic endosymbionts, organic matter degradation, metal utilization, and eutrophication response [6,7,8,9,10,11,12].

With the development of genome decoding techniques and high-throughput sequencing technologies, microbial community proteomics has quickly emerged over the past few years [13]. Much effort has been devoted to the microbial community proteomics in a variety of environments, including marine water [14,15,16,17], soils [1,18,19], sediments [8,9], activated sludge [20,21,22], groundwater [23], and acid mine biofilms [24,25]. These studies provide new insights into the outcome of gene expression regulation, protein synthesis, and the stability and turnover of mRNA and protein in response to environmental stress at a given time [26]. Moreover, these functional dimensions of the environmental proteomic database have facilitated the link of the individual microbial species to its ecological function [27]. In contrast to methods such as stable isotope probing [28], fluorescence in situ hybridization with microautoradiography [29], and full-cycle rRNA analysis [30], metaproteomics can reflect physiological activity and translational regulation of microbes in various environmental conditions. In this review, we highlight the advances of microbial community proteomics in the context of marine and freshwater, soil and sediment, activated sludge, acid mine drainage (AMD) biofilms as well as symbiotic communities. The challenges and perspectives of this field are also discussed.

## 2. Strategies for Microbial Community Proteomic Studies

**Figure 1:**
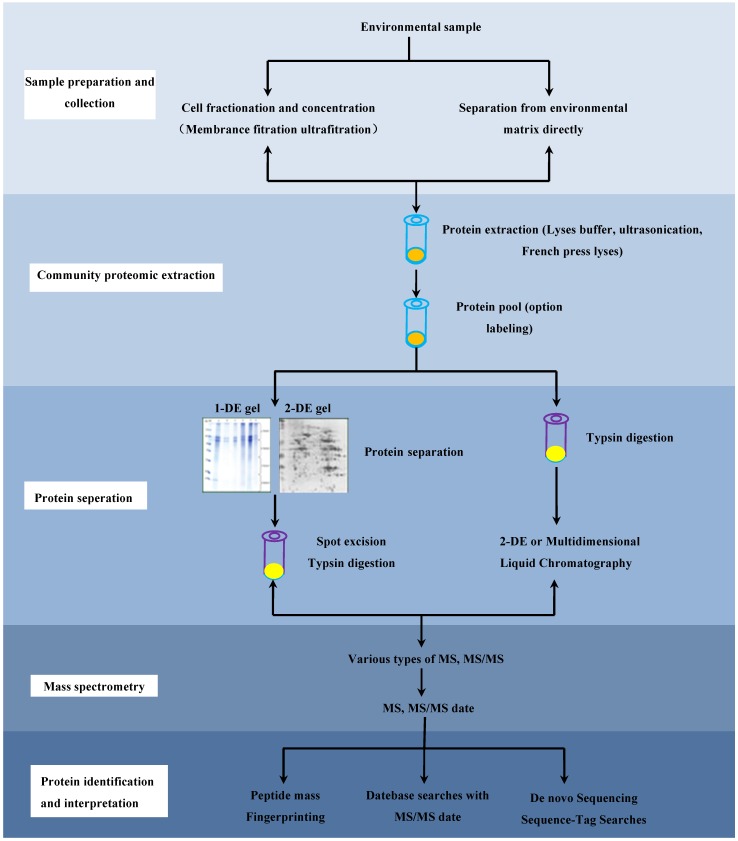
Typical workflow for microbial community proteomic analysis.

In the past few years, much effort has been devoted to exploring the strategies for microbial community proteomic studies, and several typical steps have been developed, including sample collection, protein extraction, protein separation and/or fractionation, mass spectrometry analysis, database searching and finally data interpretation (Figure 1). Because of the complex nature of environmental samples, specific approaches for either sample collection or protein extraction are developed when dealing with marine and freshwater as well as soil samples [31,32,33]. For protein separation and identification, two strategies have been established: one is the gel-based method. Traditionally, mixed proteins are separated using either one-dimensional or two-dimensional polyacrylamide gel electrophoresis (2-D PADE). Then, the target protein spots or bands are excised and proteins are digested into peptides with trypsin or other enzymes. Subsequently, the resulting peptides are subjected to mass spectrometry (MS) or tandem MS (MS/MS) analysis, database searching and bioinformatic analysis [5]. The other strategy is the liquid chromatography (LC)-based method, where the whole proteome is digested into a more complex peptide mixture using proteases without prior protein separation in gel. Then the resulting peptides are separated using strong cation exchange chromatography or microcapillary reverse-phase. In general, the separated peptides are analyzed using liquid chromatography coupled with MS/MS (LC-MS/MS). The produced MS data are interpreted for protein identification and then bioinformatic analysis. The second approach circumvents the limitations of the gel-based approach, and greatly increases the proteome coverage compared with the gel-based method, allowing high-throughput identification of thousands of proteins within a short time [34] and especially making detection of insoluble membrane proteins possible [35]. Thus, the LC-based approach has become the main stream of microbial community proteomic studies, although it still suffers from problems of reproducibility, dynamic range, and database availability. Reproducibility of metaproteomic analysis is critical to determine whether the variation of protein expression in the microbial community is environmentally relevant or the result of system errors. Usually, the technical reproducibility can be close to 50% across triplicates and over 67% between replicates using the same MS platform [36]. However, it should be noted that more replicates might improve the protein identification but the reproducibility may become worse, especially for the biological repeats.

Besides qualitative analysis in proteomics, the output of a large scale of quantitative information with high reproducibility and accuracy is rather useful, especially for comparative and quantitative proteomics: their main goal aims at determining the differences in protein expression among different biological states (e.g., control vs. treatment, healthy vs. disease, specific genotype vs. wild type) or along environmental gradients (e.g., nutrient and salinity gradients). Recently, different labeling techniques for proteomics have been developed, such as stable isotope labeling using amino acids in cell culture [37], tandem mass tags [38], stable isotope-labeled peptides [39], isotope dilution [40], isotope-coded affinity tags [41] and, more recently, isobaric tags for relative and absolute quantification [42]. However, most label-based quantification approaches are limited in complex sample preparation, protein enrichment and incomplete labeling as well as in number of samples. With the development of suitable computational software, a label-free quantitative proteomic approach has emerged, which allows the profiling of a large scale of proteins with the flexibility of multiple different comparisons. The label-free method is a semi-quantification based on the comparison of either the peak intensity of the same peptide or the spectral count of the same protein, and abundant proteins produce more spectral counts or peptide intensities. In addition, it is cost-effective due to its non-labeling characteristic. As a result, the MS-based label-free approach has been more popular and has become the main research method in metaproteomics (Table 1).

## 3. Microbial Community Proteomics in Various Environments

### 3.1. Marine and Freshwater Metaproteomics

As the Earth’s largest aquatic ecosystem, the marine habitat harbors diverse microbial communities which play a central role in regulating biogeochemical cycling of biogenic elements, including carbon, nitrogen and phosphorus, as well as various micronutrients and trace metals [68]. Deciphering metabolic activity and the ecosystem functioning of specific microbial assemblages in a variety of marine habitats provides new insights into carbon cycling as well as nutrient and energy utilization in the ocean. Since the pioneering metaproteomic work on the marine microbial community reported by Kan et al. [14], more efforts have been devoted to metaproteomic studies on the marine microbial communities.

Marine microbes can adapt to different nutrient environments through expressing abundant transporter proteins with ATP binding cassette (ABC)-type and tripartite ATP-independent periplasmic (TRAP)-type being the most abundant [69]. Similar results are reported in a membrane quantitative metaproteomic study from the South Atlantic Ocean in which TonB-dependent transporters dominate the membrane proteins [43]. A quantitative proteomic investigation of the microbial community in the coastal northwest Atlantic Ocean is also characterized by the prevalent periplasmic-binding proteins (PBPs) of ABC transporters (751 proteins) and TRAP transporters (202) [15]. The proportion of transporters shows a seasonal variation, more obviously at the deep layer (from 17% in winter to 57% in spring), indicating fierce competition within the microbial community of deep waters in spring, when organic compounds (i.e., sugar, amino acids, taurine, dipeptides and glycine betaine) are replenished owing to phytoplankton production. It is interesting that approximately 91% of the transporter spectra belong to the SAR11 and *Rhodobacterales* clades, which is consistent with the abundance of the SAR11 clade throughout the ocean, especially in oligotrophic water as well as in the bathypelagic region [68]. In a recent study, most of the ABC-type sugar-, organic polyanion-, and glycine betaine-transport proteins are identified from *Pelagibacter*, indicating their important roles in marine carbon and nitrogen cycling [44].

In order to evaluate the microbial response to nitrogen limitation in the Pacific Ocean, targeted metaproteomics is applied to investigate the protein expression profiles of the major phytoplankton groups [45]. In this study, a specific peptide biomarker for nitrogen response regulator NtcA is identified abundantly in the oligotrophic region of the North Pacific, which was consistent with the prevalence of the *Prochlorococcus* urea transporter proteins (UrtA) in low-nitrogen areas. The *Roseobacter* clade contributes a large portion of the ABC transporter (13.7% of the total metaproteome) for amino acids and polyamines, suggesting that the *Rosebacteria* rely on these nitrogen-containing organic matters [46].

SAR11 is the dominant group of α-*proteobacteria* throughout most sections of the ocean, and its adaption to oligotrophic environments has attracted great attention. A large number of mass spectra disproportionately map the periplasmic substrate-binding proteins (PSPs) from SAR11; for example, two PSPs for phosphonate acquisition are the most frequently detected, suggesting the active expression of the phosphorus transporter of SAR11 in response to phosphorus limitation in the Sargasso Sea [70]. However, in another distinct ecosystem, a productive coastal upwelling system, the highly detected transporter proteins from SAR11 are involved in amino acid, taurine and polyamine transport, as well as highly abundant glutamine synthetase [46], which is in accordance with the nitrogen and carbon limitations in this region.

In addition to the accumulation of transporters with a high affinity of nutrients, microorganisms have evolved distinct metabolic strategies to utilize hydrogen [16], one-carbon compounds [47], urea [48,71] and taurine [46], as well as other potential substrates as energy sources. A semi-quantitative metaproteomic analysis of the dissolved organic matter (DOM) from the surface and bathypelagic layers of the South China Sea indicates that the most abundant protein at the surface is the urea ABC transporter, whereas methylene tetrahydomethanopterin reductase dominates the proteome of the abyssal small-size fraction of DOM, suggesting that microbes can utilize urea as an alternative nitrogen source in the oligotrophic surface water [48]. Proteins involved in two chemolithoautotrophic pathways, the 3-hydroxypropionate/4-hydroxybutyrate cycle and the reverse tricarboxylic acid cycle, dominate the winter metaproteome of cold and dark polar water in the Western Antarctic Peninsula [46]. Consistent with the chemosynthesis cycle, ammonia is oxidized to available nitrate by the archaea and bacteria through ammonia monooxygenase. The genes of two ammonia-oxidizing *Betaproteobacteria*-associated RuBisCO enzymes are also detected in the winter metagenome. In addition, transporters and enzymes participating in taurine uptake and degradation, including taurine-pyruvate aminotransferase and sulfoacetaldehyde acetyltransferase, are abundantly detected, suggesting their important roles in regulating carbon and nitrogen utilization in the deep dark sea. The study of microbial communities from nutrient-enriched coastal systems shows the large subunit of methanol dehydrogenase from the OM43 clade in almost all the samples [47]. In a proof-of-concept experiment, the RuMP cycle is regarded as the main carbon assimilation pathway in the *Methylophaga*-like bacterium [49]. Moreover, hexulose-6-phosphate synthase, the key enzyme of the RuMP pathway of OM43, is also detected in all Atlantic Ocean samples. In addition, methanol oxidation proteins originating from the common OM43 marine clade are also identified in a deep and stratified estuary [50]. These results support the in situ activities of the OM43 clade using one-carbon compounds for energy production. Recently, Kleiner et al. combined metaproteomic quantification and metabolomic technologies to reveal that chemosynthetic symbionts can utilize carbon monoxide (CO) which has been previously thought to be unavailable for microbial nitrification due to its toxicity to aerobic organisms [16]. However, both aerobic and anaerobic CO dehydrogenases are detected in three types of *Olavius algarvensis* symbionts, indicating that they could utilize CO produced in the sediment at the sampling site. In addition, the identification of periplasmic uptake (NiFeSe) hydrogenases assigned to the δ-symbionts in the counterpart metaproteome demonstrates that energy production from hydrogen occurs in the sulfate-reducing symbionts. Notably, 544 previously unassigned proteins in the metagenomic analysis are annotated to a specific symbiont based on proteomics-based binning. Therefore, complementary information of the symbiotic community is obtained using the combined genomic and proteomic approaches, including the utilization of CO, sulphur and hydrogen in a certain specific symbiont. However, proteomic information of one symbiont, the spirochete, is completely missing owing to the lack of unambiguous metagenomic annotation for this species. Overall, phylogenetic analysis based on proteomics depends on genomic information. However, proteomic-based binning after the enrichment of a microbial group, to some extent, may overcome the obstacle and provide further functional insights.

Trace metals are essential nutrients needed for bacteria to survive on the Earth, and metalloproteins play vital roles in catalyzing critical biogeochemical reactions [72,73]. Recently, metaproteomics has been applied to explore microbial adaptive strategies in metal acquisition and utilization in various environments [8,72,73]. The uptake of limiting metals is a key driver of the ongoing adaptive strategies by which microbes evolved. Either Iron (Fe) or zinc (Zn) could form the active center of alkaline phosphatases. Therefore, they are two essential metals involved in phosphate acquisition by microorganisms. When low phosphate is available, two types of alkaline phosphatase enzymes, PhoA and PhoX, collaboratively function based on the availability of Zn or Fe. Another study reveals that the flavodoxin protein, which is the equivalent alternative of the Fe-binding protein, is abundantly distributed in the low-Fe waters in the Pacific Ocean. Unlike numerous studies that focused on well-oxygenated oceanic waters and special microbial metalloproteomes [73], Glass et al. explored microbial metal utilization in a deep-sea methane seep ecosystem using the metaproteogenomic approach [8]. Their results indicate that the anaerobic oxidation of methane bacteria can produce nickel-binding ligands to release nickel from HS^−^ outside the cells so as to increase nickel availability, which thereafter is captured by Ni-bound ligands. Similar to nickel, cobalt exists mainly in the form of Co(HS)_2_, which is less bioavailable for microbial cells. To deal with this, microbial consortia can produce high-affinity cobalt-binding ligands for acquiring the inaccessible forms. At the functional level, metaprotemics has improved our knowledge of nutrients and carbon utilization in the ocean, by providing notable information including dominant groups, transporter proteins and key enzymes involved in biogeochemical cycling.

Extreme stress environments, including hypoxia, low-light intensity and polar regions, greatly challenge microbial survival. Therefore, microbes evolve specialized strategies, i.e., sulfur oxidation and syntrophic associations, to overcome these challenges. Recently, proteins of SUP05 related to sulfur oxidation were identified, suggesting that SUP05 is able to utilize reduced sulfur compounds, such as thiosulfate or elemental sulfur (S_0_), as an energy source in the hypoxic bottom water of the Northwest Atlantic Ocean [15]. In a green sulfur bacteria (GSBs)-dominated community in Ace Lake of Antarctica [51,52] many Chlorobia-like chlorosome envelope proteins were identified using metaproteogenomics, indicating that GSBs have the ability of light capture at a high efficiency which allows them to adapt to low-light conditions. Moreover, the GSBs may facilitate their essential metabolism through coupling carbonic fixation and sulfide oxidation in the Antarctic, given that many proteins related to the sulfur cycle, such as the dissimilatory sulfite reductase system, a polysulfide-reductase-like complex, as well as a number of sulfur metabolic proteins, are detected, implying this important adaptive mechanism of GSBs to sulfur-rich polar environments. However, a comparative analysis of Lake Cadagno in Switzerland [33], where the community is dominated by the GSB *Chlorobium clathratiforme*, reveals that proteins participating in sulfur metabolism are two-fold less abundant in the dark water column; therefore, the sulfur cycle is probably not active in this dark deep water. The metaomics study of Urich et al. suggests that microorganisms in deep-sea venting sediments are fueled by chemically fixed energy to maintain growth [53]. The dominant genera *Sulfurimonas* and *Sulfurovum*, as the primary producers of the upper sediment layers, can utilize H_2_S to drive CO_2_ fixation. The free-living anaerobic methanotrophicarchaea (ANME-1) is another dominant microbial species in methane-enriched cold seep sediments which plays a major role in the sulfur cycle and the biological sinking of methane [54]. Identification of cold-adaptation proteins and key metabolic enzymes involved in the reverse methanogenesis (i.e., methyl-Coenzyme M reductase) and sulfate reduction pathways (i.e., adenylyltransferase, and adenosine 5′-phosphosulfate-reductase (AprAB)) reveals the adaptive clues of the ANME-1 community to the marine cold seep systems. These metaproteomic studies provide new insights into the adaptive lifestyle of anaerobic bacteria in the anoxic and sulfur-rich regions of the dark ocean, which advances our knowledge of microbial life in extreme stress environments.

Of the numerous adaptive strategies possessed by microbial communities, symbiotic combinations (especially between chemosynthetic bacteria and their hosts) are one of the key mechanisms in adaptation to low-nutrient and high-stress environments. In the gutless marine worm *O. algarvensis*, nutrient supply depends on its chemosynthetic bacteria, which allow it to grow well in the dark deep sea which features a high concentration of sulfide and CO_2_ [16]. In addition to expressing high-affinity transporters and utilizing alternative energy sources such as CO and hydrogen to maintain normal growth under stress conditions, significant activities of active mobile genetic elements are also found [9]. Through increasing transposase profiles (the enzyme-catalyzing movement of genetic elements), host-restricted bacteria experience an evolutionary adaptation process to rapidly changing environments.

### 3.2. Soil Metaproteomics

Soil covers almost all of the terrestrial regions and harbors the most abundant and diverse microbiota on Earth, which make it into another complex and dynamic ecosystem. Soil microbial assemblages participate in the decomposition and transformation of soil materials, contaminant remediation, rhizospheric soils, semiarid soils, as well as the biogeochemical cycling of carbon, nitrogen and other biogenic elements [61]. Thus, qualitative and quantitative assessment of the protein complement in the soil environment might provide new insights into the interactions between microbes and the environment.

Semiarid soils are composed of different soil carbon contents, vegetative communities and microbial communities. The quantitative metaproteomic approach has been applied to evaluate the functional and phylogenetic information regarding semiarid soils with distinct edaphic properties and degradation levels [18]. Proteins are identified which have the potential to participate in the biogeochemical cycling of elements as well as in the oxidation of organic matter in semiarid soils, i.e., a wide variety of dehydrogenases. Proteins involved in nitrogen cycling in semiarid soils are also identified, particularly proteases and peptidases, as well as enzymes directly participating in nitrogen fixation and nitrification. In contrast to poor soils, proteins related to phycocyanin and photosystemic apoproteins from diverse cyanobacteria are identified while superoxide dismutase and catalase are detected in a majority of semiarid soils. With regard to carbon cycling, the CO dehydrogenase and several hydrolases are also identified from *Singulisphaera acidiphila* (Planctomycete). Similarly, cyanobacteria plays important ecological roles in carbon fixation during soil erosion since multiple N-metabolic proteins are identified in semiarid areas [57].

Microbial decomposition of senesced-leaf litter plays an important role in the carbon and other nutrient cycling of terrestrial ecosystems [74]. The quantitative metaproteomic analysis of beech leaf litter indicates that environmental factors including nutrients influence the structure and function of decomposers during decomposition [58]. Fungi are the major producers of extracellular hydrolytic enzymes while no bacterial hydrolases have been detected. The litter nutrient content and stoichiometry affect microbial succession, together with decomposer community structure and activity. Moreover, microbial activity is stimulated by high litter nutrient content via high expression and high activity of extracellular enzymes.

Rhizospheric microbes are another hot spot in the terrestrial research field that aims at uncovering the interactions between plants and microorganisms in the soil ecosystem. Plant root exudates significantly affect the diversity of the microbial community. Conversely, rhizospheric microbes provide a multitude of benefits to their host including promotion of plant growth, stimulation of pathogen resistance or direct defense against pathogens as well as nutrient supply [74]. Wang et al. characterizes the metaproteomes of different crop rhizospheric soils (CRS) using 2-DE coupled with MALDI-TOF/TOF-MS [19]. Among the successfully identified 189 protein spots, 72 derived from the microflora are involved in protein, energy, nucleotide and secondary metabolism, as well as signal transduction and resistance. Most of these biological processes are associated with the soil nutrient cycle, particularly carbon and nitrogen. These proteins might play crucial roles in the communication among plants, microbes and fauna, and induce metabolic changes inside the organisms. A comparison of the CRS subjected to increasing periods of *Rehmannia glutinosa* reveals that the identified proteins derived from plants and microorganisms actively participated in nutrient assimilation and energy transformation in the rhizospheric soil ecosystem [59]. They participate in protein, nucleotide and secondary metabolism, signal transduction and resistance, and 33 differentially expressed protein spots are shown to respond to an increase in the monoculture years. Among them, most of the upregulated plant proteins are involved in carbon and nitrogen metabolism and stress response, while the majority of the upregulated microbial proteins participate in protein metabolism and cell-wall biosynthesis. With an increase in the monoculture years, the phenylalanine ammonia-lyase significantly increases in total phenolic acid content, implying that it participates in the phenylpropanoid metabolism. These studies indicate that the consecutive monoculture of *R. glutinosa* changes the soil microbial ecology owing to the accumulation of exudates, which in turn might affect the nutrient cycle, resulting in plant growth and development retardation.

The metaproteomic approach has also been employed to characterize microbial metabolic activities relevant to the bioremediation of pollutant-contaminated environments. Using the gel-based approach, the metaproteome of cadmium-contaminated soil is analyzed, although very limit protein information is obtained [75]. A proteomic-based study on the uranium-contaminated aquifer demonstrates the importance of the dominant *Geobacter* community members as well as their pathways involved in energy generation during biostimulation [60]. However, a recent study on the initial responses of the indigenous aquifer microbiota to biostimulation with emulsified vegetable oil at a uranium-contaminated site suggests that members of the *Betaproteobacteria* and the *Firmicutes* dominate the biostimulated aquifer community [76]. Organic pollutants in soils have also attracted great attention. Metaproteomic analysis of the microbial community from 2,4-dichlorophenoxy (2,4-D)-contaminated soils indicates that at least two species are linked to the biodegradation of chlorobenzene and that the 2,4-dichlorophenoxyacetate dioxygenase involved in 2,4-D degradation is expressed by autochthonous bacteria [23]. Recently, a culture-dependent community proteomic study traced changes in the microbial assemblies of a hydrocarbon-polluted soil [61]. The results suggest that the soil microbial community becomes more complex in hydrocarbon-polluted soil compared to that in untreated soil. Although *Bacillus* sp. dominates in both communities, other species, such as *Ralstonia solanacearum*, *Synechococcus* elongates and *Clostridium* sp., do not appear in the non-contaminated soil, suggesting their resistance to hydrocarbon contamination. A further study on the bioremediation of hydrocarbon contamination indicated that compost-assisted bioremediation is mainly driven by *Sphingomonadales* and uncultured bacteria through the high expression of catabolic enzymes such as catechol 2,3-dioxygenases, cisdihydrodiol dehydrogenase and 2-hydroxymuconic semialdehyde [62]. A similar metaproteomic survey of toluene-amended soil as well as enriched cultures containing toluene and soil extracts shows that many proteins are shared between the two toluene-amended communities [1]. Compared with glucose-amended soil as the control, a high expression of glutamine synthetase, ABC transporters, extracellular solute-binding proteins, and outer membrane proteins in both toluene-amended communities might be involved in the removal of toluene from the bacterial cells. Overall, metaproteomic approaches provide a valuable avenue to explore the roles of the major particular bacteria with specific functions in situ rather than in the traditional “artificial” laboratory experiments [77].

### 3.3. Wastewater and Activated Sludge Metaproteomics

Microbial communities play important roles in wastewater treatment and different microbial systems have been developed [78]. Metaproteomics provides an interesting functional insight into the complex microbial communities in the wastewater. Carla et al. employ the metaproteomic approach to investigate the response of an unsequenced bacterial community in a continuous-flow wastewater treatment bioreactor with an inhibitory level of cadmium [63]. The metaproteome in the bioreactor has a quick response (after 15 min) to cadmium exposure and shows a temporal change compared with the unexposed control at each time point (0.25, 1, 2 and 3 h). More than 100 unique differentially expressed proteins are identified, including ATPases, oxidoreductases, and transport proteins. Metaproteomics has also become a critical research component of activated sludge wastewater treatment. Wilmes et al. conducted a series of metaproteomic studies on the molecular mechanisms of enhanced biological phosphorus removal (EBPR) [4,21,22]. Although only several proteins are identified in their first effort, metaproteomics shows its potential in the study of activated sludge [4]. With both the improvement of the MS technique and the availability of metagenomic data, great achievements have made in the metaproteomics of the activated sludge system. Based on the gel-based proteomic approach, 46 proteins among the 111 excised spots are identified and many of them are closely matched to “Candidatus *Accumulibacter phosphatis*”, indicating that the *Accumulibacter*’s metabolic activities are related to the chemical transformations in EBPR. Furthermore, more than 700 proteins are identified from the *A. phosphatis* population using a non-gel-based proteomic approach, and these proteins are involved in many key metabolic pathways, such as denitrification, fatty acid cycling and the glyoxylate bypass, with significant importance in EBPR. The differences in protein abundance for enzyme variants related to core metabolism and EBPR-specific pathways, as well as genetic diversity, are crucial for maintaining the stable performance of EBPR systems. Park et al. investigate activated sludge extracellular proteins in sludge digestion using SDS-PAGE combined with LC-MS/MS [64]. The results suggest that activated sludge flocs contain different fractions of proteins and each fraction undergoes a different fate in anaerobic and aerobic digestion. Several bacterial proteins and sewage-derived polypeptides are identified, indicating that microbial interactions are mediated by extracellular enzymes [79]. In a recent study on the characterization of the microbial communities from continuous stirred tank reactors for digesting sewage sludge, a large number of proteins are identified as belonging to the “Candidatus *Competibacter*” group, suggesting that this microbial group play key roles in phosphorus removal [65]. Overall, meteproteomics enhances our understanding of the microbial communities and their functions in different sludge systems.

### 3.4. Acid Mine Drainage (AMD) Biofilm Metaproteomics

AMD refers to the extremely acidic (pH < 3), metal-enriched waters derived from pyritic material [80]. AMD is regarded as the principle environmental problem in the global mining industry, and the water should be treated to remove metals and raise the pH before discharge. AMD is an enormous environmental issue associated with energy and metal resources; for example, the burning of sulfur-rich coal leads to the release of contaminants such as mercury and the formation of acid rain [81]. Microorganisms associated with AMD are of great concern, having many effects on the formation, the pollutant release, and the biological remediation of AMD.

A pioneer study was conducted in 2005 using quantitative metaproteomic analysis to evaluate the in situ microbial activity of a natural AMD microbial biofilm community with low complexity [24]. In total, 2033 proteins were identified from the five most abundant microbial species and nearly half were derived from *Leptospirillum* group II. The high expression of proteins related to protein refolding and oxidative stress is regarded as a critical role for microbial survival. More excitingly, an abundant novel protein is determined to be a cytochrome central to iron oxidation and AMD formation. A similar proteogenomic strategy is employed to identify proteins in natural acidophilic biofilms [25]. With strain specificity, the proteomic results reveal a genome shaped by the recombination of two closely related bacterial populations. The confirmation of a large scale of inter-population genetic exchange indicates that this exchange is key to the adaptation to specific ecological niches partitioning the AMD biofilm. Denef et al. further analyzed the dominant *Leptospirillum* group II populations from 27 biofilms of the AMD system [66]. The results indicate that the specific environmental conditions select the particular recombinant variants, thus leading to a fine-scale tuning of microbial populations. Genes involved in motility, signal transduction and transport are over-expressed in tens to hundreds of kilobase recombinant blocks, whereas core metabolic functions are significantly down-expressed. Goltsman et al. also employ community genomic and proteomic approaches to investigate chemoautotrophic Fe-oxidizing *Leptospirillum* group II and III bacteria in AMD biofilms [67]. *Leptospirillum* groups II and III are responsible for 64.6% and 44.9% of the predicted proteins, respectively, and 20% of the proteins are identified as plasmid proteins. Among them, the proteins identified from both bacterial groups are involved in community-essential functions, including carbon fixation and the biosynthesis of vitamins, fatty acids, and biopolymers (including cellulose). Notably, these studies indicate that the AMD system is often dominated by *Leptospirillum* groups II and III. Signal transduction and methyl-accepting chemotaxis proteins are abundant in *Leptospirillum* group III, while *Leptospirillum* group III possesses a methyl-independent response pathway.

## 4. Challenges

In general, the metaproteomic approach has been widely applied to study microbial communities from various environmental circumstances in the past few decades, and it has provided new insights into microbial diversity, metabolic potential, ecological function and microbe-environment interactions. However, because of the complexity and diversity of environmental samples, this technology still faces great challenges in the study of environmental microbial communities.

To fully understand the role of microbial communities in the environment, we should obtain as wide a range of proteins or protein information as possible, especially for those low-abundance proteins. Up until now, there are still a few challenges from the technological point of view. The first challenge is sample collection and preservation. For most environmental samples, the density of the microbial population is very low, and, furthermore, the vast majority of microorganisms in the environment cannot be cultured. Various sample collection methods, such as ultrafiltration and flow cytometry sorting, have been developed, but these methods can neither separate different microorganisms in the populations nor obtain sufficient cell biomass of the different microorganism species. The protein information obtained from the metaproteome just reflects the abundant microbes in the populations but not the rare or sparse species, and this hinders our understanding of the function and role of different microbial species in the environment.

On the other hand, in order to reflect the real microbial world, the in situ environmental sample should not be altered too much. For some samples, it is convenient and flexible to use quick fixation and preservation at a low temperature, such as liquid nitrogen, immediately after sample collection. However, it could become a problem when sampling is conducted under extreme environments, for example samples from habitats under oxygen deficit, extremely high or low temperatures or high pressure. Microbial communities are very sensitive to environmental alterations and respond quickly. When it is difficult to maintain the natural conditions, it is better to cut down the time cost of sample collection. Nevertheless, it is still a problem when the microbial biomass is low and has to be enriched, or a longer time is required to transport samples, such as in the case of deep ocean samples. In a word, care should be taken to maintain microbial communities at an in situ status, which is a big challenge for microbial metaproteomic studies.

Another obstacle for microbial metaproteomics is protein extraction from complex environmental samples, which are mixtures of various organic and inorganic materials, such as humic acid, lignin, chemical chelation, cell exudation and various degradation products. Different protein extraction methods have been developed based on the features of the environmental samples, such as soils [82], sediments [54], sea water samples [15], activated sludge [83], biofilms [84], marine organic particles [85] and symbionts [16]. However, due to the heterogeneous species distribution, the wide range of protein abundance levels, and the unextractable proteins binding to the membrane or soil matrix, there is no standard and efficient protocol for extracting proteins from environmental samples [70]. Thus, it is essential to explore new extraction methods and optimize protein quantification efficiencies regarding the specific physical, chemical and biological properties of the individual sample.

As the last but most important step, the identification of proteins is crucial for microbial metaproteomic studies. Several challenges hold back protein identification, which greatly depends on the database design, capacity and quality influencing the resulting peptide sequence matches. First, peptide sequence matching against such a large database suffers from the increased potential for false-positive matches, which lowers the number of highly confident true matches [86]. The second challenge is that the confidence of protein assignments to taxa is limited by the species present in the database, and functional assignments are often therefore more robust than taxonomic assignments of proteins. The last challenge concerns the explosive increase of the protein database which makes protein identification extremely time-consuming and demanding on hardware. To improve peptide identification, metagenomic data derived from the sampling sites can be used as the reference [26]. Another approach is to combine metagenomic with metaproteomic analysis, thus providing an enhanced means to reconstruct the microbial processes of a community [8].

## 5. Perspectives

Although challenges still exist in microbial community proteomics, the improvements in protein sample preparation and downstream MS technology along with the fast growth of bioinformatics tools and various databases might overcome these limitations and speed up microbial metaproteomic research.

One of the trends of microbial community proteomics in the future will be the move from the qualitative analyses of function and activity surveys to the quantitative analyses of protein expressions and dynamics in environmental samples. Nowadays, to satisfy the demands of studies of systematic biology or biomarker discovery in environmental microbiology, it is necessary to obtain the precise quantitative information of a predefined set of proteins or all proteins in environmental samples. The most widely used technique is called selected reaction monitoring or multiple reaction monitoring (MRM) [87]. Recently, a group of protein biomarkers were reported to diagnose ocean metabolism in Pacific Ocean biomes using MRM [45], which provides a good example of a targeted proteomic study conducted in an environmental microbial community. However, this method could only target a limited number of proteins compared to a global proteomic approach. Recently, an alternative approach, the SWATH-MS technique, was introduced, in which fragment ion maps are generated using a data-independent acquisition method to give accurate global quantitative information. [87]. A proteome dataset with more accurate quantitative information combined with other omic (metagenomic and metatranscriptomic) datasets will provide an entire view of the functions, activities and interactions in real microbial communities.

Another exciting new direction is the characterization of metabolic activities and the interpretation of adaptation for a specific microbial population or species in natural environments, given that many microbes cannot be cultivated and that the metabolism of those growing in monoculture are unlikely to be the same as those of organisms growing in assemblages. Improvements of the sensitivity and accuracy of the MS technique enable the possible MS-based characterization of amino acid substitutions. A proteogeonomic study in an AMD community [25] presents the strain-resolved capability of microbial metaproteomics. The key finding of this study is the possibility of identifying peptide sequences shared with sequenced organisms when multiple genomic data sets from closely related microbes in the community are available. The high resolution of the proteomic approach is further demonstrated by another study in a more complex community of surface seawater in the Sargasso Sea [56]. Moreover, proteomics-inferred genome typing reveals an adaption strategy of *Leptospirillum* group II to environmental stress through inter-population recombination [66]. Recently, a novel sequencing approach, single-cell sequencing [88,89], was developed, which provides cell-specific genetic information from a single cell of the non-cultured bacteria, even for the low-abundance organisms. In addition, both host sequence contamination and the difficulty of metagenomic assembly can be easily bypassed [3]. Therefore, elegant combinations of both single-cell sequencing and metaproteomics will provide new insights into the function and activity via species-specific protein identifications among a diverse community. Despite the currently high costs per sample and per depth of single-cell sequencing relative to metagenomics, the emergence of this technique will greatly advance the capability of cross-strain identification in community proteomics. With a high throughput of strain-specific proteome data, it also becomes possible to investigate the post-translational modifications in in situ environments, including phosphorylation, acetylation, glycosylation, ubiquitination and glutathionylation, which are extensively used by bacteria to transmit signals and to coordinate cellular functions. For example, bacterial protein phosphorylation is considered to be a signal transduction device which mainly links environmental factors to the regulation of important physiological processes [90].

Finally, besides metaproteomics, other omic approaches, such as metagenomics and metatranscriptomics, as well as metabolomics, are still being rapidly developed to pave the way for integrated multi-omic approaches in microbiology. With the advantages of computational tools, an understanding of the systems biology of the natural microbial community is the future trend to integrate and meta-analyze multiple data sets.

## Figures and Tables

**Table 1:** Survey of metaproteomic studies in different environments from a literature search.

Environment	Subject of Analysis	Protein-Method		Major Findings	Refs/Year
		Separation	Identification		
*Marine and Freshwater*					
South Atlantic	Microbial membrane proteins from surface water	LC-MS/MS		TonB-dependent transporters dominated bacterial membrane proteins while bacterial rhodopsins were detected in every sample; *Archaeal*, ammonia monooxygenase proteins were identified in upwelling region.	[43]/2010
The Northwest Atlantic Ocean	Metabolic activity of microbial plankton in a seasonally hypoxic basin	LC-MS/MS		A seasonal increase in high-affinity membrane transport proteins involved in scavenging of organic substrates; *Rhodobacterales* transporters were strongly associated with the spring phytoplankton bloom, whereas SAR11 transporters were abundant in the underlying waters.	[15]/2014
Six disparate aquatic habitats	Microbial populations	LC-MS/MS		ABC-type sugar-, organic polyanion–, and glycine betaine–transport proteins were identified from *Pelagibacter*, and these transporters play important roles in carbon and nitrogen cycling.	[44]/2014
The intersecting Pacific Ocean	Multiple nutrients		MS-based Multiple Reaction Monitoring	Nitrogen response regulator NtcA was abundant, which was consistent with the prevalence of the *Prochlorococcus* urea transporter proteins (UrtA) in low-nitrogen areas.	[45]/2014
The Antarctic Peninsula coast	Bacterioplankton of winter and summer	SDS–PAGE	MS/MS	*Roseobacter* clade contributed a large portion of ABC transporter for amino acids and polyamines; transporter proteins involved in amino acid, taurine and polyamine transport, as well as glutamine synthetase, were highly detected in SAR11; proteins involved in two chemolithoautotrophic pathways dominated the winter metaproteome of cold and dark polar water.	[46]/2012
The Oregon coast	Microbial plankton of upwelling region	2D-LC-MS/MS		Thirty-six percent and 17% of detected proteins were from the SAR11 clade and *Roseobacter* clade; transporters for amino acids, taurine, polyamines and glutamine synthetase were highly detected; methanol dehydrogenase was detected.	[47]/2011
Symbionts	A gutless worm and its symbiotic microbial community	1D PAGE; 2D	LC-MS/MS	Sulfur oxidation proteins; aerobic and anaerobic CO dehydrogenases were detected in three types of *Olavius algarvensis* symbionts; high expression of periplasmic uptake (NiFeSe) hydrogenases and high-affinity uptake transport related proteins were detected.	[16]/2013
The South China Sea	Dissolved organic matter (DOM) from marine surface and bathypelagic region	SDS-PAGE	LC-MS/MS	*Archaea* and *Proteobacteria* were the major contributors to bathypelagic proteome; protein compositions differed along the vertical water column, and urea ABC transporter was abundant in the surface DOM.	[48]/2011
The English Channel	Natural populations	Protein-SIP LC-MS/MS		RuMP cycle was the main carbon assimilation pathway in *Methylophaga*-like bacterium, and methanol dehydrogenase–encoding gene mxaF, as well as three out of four identified xoxF homologues were expressed.	[49]/2015
The Lower St. Lawrence Estuary	Microbial communities through the stratified water column	LC-MS/MS		Chemosynthetic production coupled to nitrification by MG-I *Thaumarchaeota* and *Nitrospina* was a dominant metabolic strategy; methanol oxidation proteins were detected from the OM43 marine clade; membrane transport proteins were assigned to the uncultivated MG-II *Euryarchaeota*.	[50]/2015
Sulphidic marine sediments	Trace metal utilization of methane-oxidizing microbial consortia	LC-MS/MS		Microbial consortia relied on the nickel metalloenzymes and transporters, cobalt metalloenzymes and transporters, molybdenum and tungsten enzymes to catalyze anaerobic oxidation of methane (AOM).	[8]/2014
Ace Lake in Antarctica	Green sulfur bacteria	SDS-PAGE	LC-MS/MS	Proteins that participated in DNA processing, nucleic acid binding, folding/refolding of proteins and lipid biosynthesis were identified to be involved in cold adaption of green sulfur bacteria.	[51]/2010 [52]/2011
The meromictic Lake Cadagno	Green sulfur Bacterium *Chlorobium clathratiforme*	LC-MS/MS		*Chlorobium clathratiforme* contained enzymes for fixation of N(2) and oxidation of sulfide to sulfate, and they were not active in the dark; fermentation of polyglucose in the dark was the major pathway to obtain energy.	[33]/2011
Hydrothermal venting sediments	Microbial community structure and functioning	SDS-PAGE	LTQ Orbitrap-MS/MS	Epsilonpro-teobacteria, δ- and γ-proteobacteria, ciliates, nematodes and various archaeal taxa were identified; high expressions of carbon fixation pathways as well as chemotaxis and flagella genes.	[53]/2014
Marine seep sediments	Free-living ANME-1; Sulfate-reducing bacteria	2-DE	MS	Anaerobic methanotrophic archaea dominated microbial species involved in the sulfur cycle and the biological sinking of methane; cold-adaptation proteins and key metabolic enzymes involved in the reverse methanogenesis and sulfate-reduction pathways were identified.	[54]/2012
Symbionts	Microbial community of the sponge *Cymbastela*	LC-MS		Proteins involved in cold adaptation and production of gas vesicles were abundant; high expressions of affinity transporters and alternative energy–utilizing proteins under stress conditions.	[55]/2012
The Sargasso Sea	Microbial membrane proteins of surface water; the SAR11 clade	LC-MS/MS		SAR11 periplasmic substrate-binding proteins (PBP) for phosphate were most abundant; proteins involved in amino acids, phosphonate, sugars and spermidine were detected.	[56]/2009
The western South China Sea	Particulate organic matters (POM) from marine surface and mesopelagic layers	SDS-PAGE	LC-MS/MS	Cyanobacteria was the largest contributor; photosynthesis-associated proteins; porins, adenosine triphosphate synthases, nutrient transporters, molecular chaperones, and ectoenzymes were detected.	[32]/2010
*Soils*					
Semiarid soils	Functional and phylogenetic information	SDS-PAGE	LC-MS-MS	Three protein extraction methods were examined, and the functional, phylogenetic and bio-geochemical information obtained by three methods in semiarid soils presented distinct edaphic properties.	[18]/2014
Semiarid soils	Deforestation fosters bacterial diversity and the cyanobacterial community	SDS-PAGE	LC-MS-MS	Deforestation increased bacterial diversity in semiarid ecosystems and raised the abundance of cyanobacterial proteins involved in C-fixation in semiarid areas.	[57]/2015
Beech leaf litter	Environmental factors and nutrients on the decomposer structure and function	SDS-PAGE	LC-MS/MS	Fungi were the main producers of extracellular hydrolytic enzymes, and microbial activity was stimulated at a higher litter nutrient content via a higher abundance and activity of extracellular enzymes.	[58]/2012
Crop rhizospheric soil	Crop soil metaproteomics	2-DE	MALDI-TOF/TOF-MS	Proteins involved in protein, energy, nucleotide, secondary metabolisms and signal transduction and resistance were identified; most upregulated plant proteins were involved in carbon and nitrogen metabolism and stress response, while the majority of the upregulated microbial proteins participated in protein metabolism and cell-wall biosynthesis.	[19]/2011 [59]/2011
Toluene-amended soil	The microbial community proteome	SDS–PAGE	MALDI-MS	Glutamine synthetase (Gln), ABC transporters, extracellular solute-binding proteins, outer membrane proteins (Omp) were upregulated in toluene-amended soil; arginine deiminase (ArcA) and cold-shock protein presented in toluene-amended culture while superoxide dismutase (SodB) and chaperonin (GroEL) presented in toluene-amended soil.	[1]/2010
Humic soil	Enzymes connected with bacterial metabolic pathways	SDS-PAGE; 2-DE	LC-ESI-MS	Protein extraction method from soil was developed, and 2,4-dichlorophenoxy acetate dioxygenase, chlorocatechol dioxygenases, molecular chaperons and transcription factors were identified.	[23]/2007
Uranium-amended soil	The subsurface microbial communities	2-DE	LC-MS-MS	The proteome was dominated by the enzymes converting acetate to acetyl-coenzyme A and pyruvate for central metabolism. *Geobacter* dominated the microbial community and they participated in energy generation during biostimulation.	[60]/2009
Hydrocarbon-polluted soil	Changes in the microbial community	SDS-PAGE	HPLC-MS/MS	The complexity of the microbial community showed a relative increase in hydrocarbon-enriched cultures, and the majority of identified proteins were related to glycolysis pathways, structural or protein synthesis.	[61]/2010
Hydrocarbon-polluted soil	Changes in the microbial community	SDS-PAGE	LC-MS/MS	*Proteobacterial* protein expressions increased while the abundance of *Rhizobiales* decreased during petroleum pollution; compost-assisted bioremediation was mainly driven by *Sphingomonadales*; abundances of catechol 2,3-dioxygenases, cis-dihydrodiol dehydrogenase and 2-hydroxymuconic semialdehyde were increased.	[62]/2016
*Wastewater and Activated Sludge*					
Cadmium-polluted wastewater	The response of a natural community	2-DE	MALDI-TOF/TOF MS	Significant community proteome responses to cadmium exposure were observed, and ATPases, oxidoreductases, and transport proteins played important roles in the cadmium shock.	[63]/2007
Wastewater sludge	Laboratory wastewater sludge microbial communities	2-D PAGE	MALDI-TOF-MS	Substantial differences in protein abundance for enzyme variants were uncovered among the *A. phosphatis* population, and these proteins were mainly involved in core metabolism, EBPR-specific pathways, energy generation.	[4]/2004 [21]/2008 [22]/2008
Wastewater activated sludge	Extracellular proteins in sludge digestion	SDS-PAGE	LC-MS/MS	The proteins resistant to degradation and generated during anaerobic digestion were identified, including a limited number of bacterial and human polypeptides.	[64]/2008
Sewage sludge	Different proteins in two parallel anaerobic digestion lines	SDS-PAGE	LC-MS/MS	Protein-inferred and 16S rDNA tags–based taxonomic community profiles were not consistent, and a high proportion of proteins belonged to “Candidatus *Competibacter*” group.	[65]/2015
*Acid Mine Drainage Biofilm*					
Acid mine drainage biofilm(AMD)	Gene expression, identified key activities, examined partitioning of metabolic functions	2-DE	LC-MS/MS	Half of the predicted proteins from the dominant biofilm organism *Leptospirillum* group II and protein involved in refolding and oxidative stress response presented high expressions; cytochrome played a central role in iron oxidation and AMD formation.	[24]/2005
Acid mine drainage biofilm	Biofilms growing at the liquid-air interface	2-DE	LC-MS/MS	*Leptospirillum* groups II and III dominated AMD system; signal transduction and methyl-accepting chemotaxis proteins were abundant in *Leptospirillum* group III, while *Leptospirillum* group III possessed a methyl-independent response pathway.	[66]/2009 [67]/2009
